# Preoperative Cryoneurolysis in Total Knee Arthroplasty: Current Concepts in Pain Control and Opioid Stewardship

**DOI:** 10.7759/cureus.106522

**Published:** 2026-04-06

**Authors:** Benjamin Hershfeld, Ryan M Marra, Adam Bitterman, Randy Cohn

**Affiliations:** 1 Orthopaedic Surgery, Northwell Health, New Hyde Park, USA; 2 Orthopaedic Surgery, Northwell Health - Huntington Hospital, Huntington, USA; 3 Orthopaedic Surgery, Donald and Barbara Zucker School of Medicine at Hofstra/Northwell, Hempstead, USA; 4 Orthopaedic Surgery, Nasuti College of Osteopathic Medicine, Duquesne University, Pittsburgh, USA; 5 Orthopaedic Surgery, Northwell Health - Long Island Jewish Valley Stream, Valley Stream, USA

**Keywords:** cryoneurolysis, multimodal analgesia, opioid stewardship, postoperative pain, total knee arthroplasty

## Abstract

Total knee arthroplasty (TKA) is increasingly common, yet postoperative pain remains a major driver of delayed recovery and opioid exposure. Cryoneurolysis is a preoperative, non-opioid adjunct that temporarily ablates peripheral sensory nerves to reduce pain signaling. This narrative review summarizes the mechanism, clinical evidence, and practical implementation of cryoneurolysis for perioperative pain control and opioid reduction after TKA.

Published clinical studies evaluating preoperative cryoneurolysis in patients undergoing TKA or with end-stage knee osteoarthritis in perioperative pathways were narratively synthesized, including retrospective analyses, cohort studies, randomized or sham-controlled trials, registry studies, and relevant systematic reviews and meta-analyses. Comparative perioperative analgesic literature relevant to multimodal TKA protocols was also considered. The review was organized around three domains: (1) biologic mechanisms and technical targets, (2) outcomes related to pain, opioid consumption, and length of stay, and (3) opioid stewardship endpoints, including refill requirements and prolonged opioid use, when available.

Cryoneurolysis produces reversible axonotmesis via controlled cooling, inducing Wallerian degeneration while preserving nerve architecture and motor function. Across published studies, cryoneurolysis was associated with reduced acute postoperative pain and lower early opioid use, with some studies reporting approximately 45% to 51% reductions in opioid consumption and shorter hospital length of stay. Evidence on postdischarge opioid utilization is mixed, with one retrospective cohort finding no difference in 90-day opioid consumption or refill rates, while other registry and longitudinal data suggest possible reductions in longer-term opioid use and improved pain outcomes, although functional benefits appear modest and inconsistently demonstrated. Available studies have not identified a clear increase in complications, and the motor-sparing nature of cryoneurolysis may support early rehabilitation.

Preoperative cryoneurolysis is a promising adjunct within multimodal TKA analgesia, offering prolonged sensory analgesia without motor blockade and short-term reductions in pain and inpatient opioid use. Heterogeneous protocols and limited long-term, high-level evidence constrain broad adoption. Standardized multicenter trials with pharmacy-based opioid endpoints are needed to define optimal targets, timing, and durable benefit.

## Introduction and background

Total knee arthroplasty (TKA) is one of the most common surgical interventions for advanced knee osteoarthritis [1]. In the United States, over one million primary TKAs are performed annually, and procedural volume is projected to continue increasing substantially in the coming years [2]. Yet, postoperative pain remains a significant clinical challenge and an important determinant of patient satisfaction and functional recovery [3]. Historically, opioid analgesics were a cornerstone of post-TKA pain management, and postoperative opioid use after TKA has been substantial [4,5]. However, opioid use carries well-documented risks, including dependence, adverse effects, and contribution to the ongoing opioid epidemic [5]. In response, multimodal opioid-sparing strategies have become the standard of care for TKA pain management, combining systemic, regional, and local modalities to reduce reliance on narcotics [6,7]. Accordingly, interest has grown in non-opioid analgesic adjuncts aimed at reducing opioid exposure while maintaining effective pain relief [6,7].

Cryoneurolysis has emerged as a promising preoperative intervention that uses controlled cold to temporarily disrupt peripheral sensory nerve signaling and reduce pain transmission [8]. Unlike traditional local anesthetic nerve blocks, cryoneurolysis is intended to provide longer-acting sensory analgesia without prolonged motor blockade. By reducing nociceptive input before surgery, cryoneurolysis has the potential to decrease postoperative pain and diminish perioperative opioid requirements. While early studies have demonstrated short-term analgesic benefit of cryoneurolysis in TKA, evidence regarding opioid refill rates and longer-term opioid use after discharge remains limited and mixed. This current concepts review summarizes (1) the mechanism of action of cryoneurolysis, (2) clinical evidence, and (3) practical applications of preoperative cryoneurolysis in TKA, with particular focus on its role in reducing postoperative pain and opioid consumption.

## Review

Mechanism of action

Cryoneurolysis is a form of targeted neuromodulation that uses extremely low temperatures to achieve a temporary interruption of peripheral nerve function. By applying a localized freezing probe to a sensory nerve, the technique induces Wallerian degeneration of axons while preserving the nerve’s connective tissue architecture [9]. This controlled cold injury disrupts pain signal transmission without causing permanent structural damage, allowing axonal regrowth at approximately 1 to 2 mm per day, with structural and functional recovery typically occurring over three to six months [10-12].

In the context of TKA, cryoneurolysis is typically performed preoperatively using a percutaneous probe (often under ultrasound guidance) to target the sensory nerves that innervate the anterior knee, most commonly the infrapatellar branch of the saphenous nerve and the anterior femoral cutaneous nerve. Ultrasound-guided cryoneurolysis provides prolonged, site-specific analgesia without local anesthetic-related motor blockade, which may offer practical advantages relative to some traditional peripheral nerve block strategies [10,13]. By inducing reversible axonotmesis in targeted sensory nerves, the technique produces an extended analgesic effect lasting several weeks without the need for continuous anesthetic infusion or indwelling catheters [13]. The treatment is generally administered several days to a few weeks before surgery, although the optimal timing can vary based on clinical protocol [8]. This lead time allows adequate axonal degeneration and diminished nociceptive input by the day of surgery.

In practice, cryoneurolysis systems are designed to generate temperatures within the range associated with reversible axonotmesis and Wallerian degeneration, typically between -60°C and -100°C, while sparing the endoneurium and other supportive nerve structures, although the exact probe temperature and treatment parameters may vary by device and protocol [14,15]. Notably, temperatures below -100°C are avoided to prevent permanent neurolysis of the nerve fiber [14]. By selectively freezing the target nerves to this critical temperature range, cryoneurolysis preemptively desensitizes the pain pathways most involved in TKA, aiming to reduce perioperative pain intensity and, consequently, lower opioid requirements.

Clinical evidence

Analgesic Efficacy - Early Postoperative Outcomes

A growing body of clinical evidence suggests that preoperative cryoneurolysis can meaningfully reduce pain and opioid consumption after TKA, although results have varied across studies. One of the earliest reports was a 2016 cohort study in which preoperative cryoneurolysis of the anterior femoral cutaneous nerves significantly reduced postoperative pain scores and opioid requirements compared to controls [16]. Patients who received cryoneurolysis in that study used 45% less cumulative morphine equivalent narcotics during the 12 weeks following surgery than controls [16]. Building on these findings, a larger 2021 study evaluated cryoneurolysis targeted to the superficial genicular nerves and reported similarly positive outcomes, including 51% lower daily in-hospital morphine milligram equivalent use, 22% lower mean in-hospital pain scores, and a 44% reduction in overall hospital length of stay relative to controls [17].

However, not all studies have shown such positive benefits. One retrospective study of 183 patients reported no measurable opioid-sparing effect following preoperative cryoneurolysis [18]. A recent systematic review and meta-analysis pooled the available comparative studies and found short-term benefits of preoperative cryoneurolysis, including reduced acute postoperative pain, lower opioid consumption, and shorter hospital stay after TKA. The pooled length-of-stay effect was modest, with a mean reduction of 0.63 days, and should be considered in the context of heterogeneity across studies and the large effects reported in some retrospective cohorts [19]. Overall, no increase in complications was identified [19]. Registry data have also suggested possible benefit beyond the immediate postoperative period, although those findings are distinct from the pooled short-term evidence [20]. Additionally, one retrospective cohort study reported that 6% of patients in the cryoneurolysis group required two or more days of hospitalization, compared with 67% of controls, although this large difference should be interpreted cautiously, given the study’s nonrandomized design and historical control group [16]. The available human clinical studies evaluating perioperative cryoneurolysis in TKA are summarized in Table 1.

**Table 1:** Human clinical studies evaluating perioperative cryoneurolysis for total knee arthroplasty Studies included: Mihalko et al. (2021) [8], Dasa et al. (2016) [16], Urban et al. (2021) [17], Wickline et al. (2024) [18], Ng et al. (2025) [20]. ITT: intention-to-treat; KOOS JR: Knee Injury and Osteoarthritis Outcome Score, Joint Replacement; LOS: length of stay; RCT: randomized controlled trial

Design	Study Years	Sample Size	Nerves Targeted	Timing	Comparator	Primary Outcomes	Key Findings	Major Limitations
Single-center RCT [8]	2017-2019	62 cryoneurolysis vs 62 controls (intention-to-treat)	Superficial genicular nerves	3-7 days preoperatively	Standard multimodal care	Cumulative opioid use through 6 weeks	Primary endpoint not met in ITT; opioid reduction in per-protocol analysis; improved early pain/function	Failed ITT endpoint; per-protocol exclusions; single-center study
Retrospective cohort [16]	2011-2014	50 cryoneurolysis vs 50 controls	Infrapatellar branch of the saphenous nerve; anterior femoral cutaneous nerve	~5 days preoperatively	Historical standard multimodal care	LOS; opioid use; pain and function	Reduced early opioid use and shorter LOS compared with controls	Retrospective design; historical controls; selection bias
Prospective cohort [17]	2019-2020	169 cryoneurolysis vs 98 controls	Superficial genicular nerves	~9 days preoperatively	Standard multimodal care	Pain scores; opioid use; LOS	Lower early pain scores, opioid use, and LOS	Non-randomized; incomplete reporting; selection bias
Retrospective cohort [18]	2022-2023	183 cryoneurolysis	Anterior femoral cutaneous nerve; inferior branches of the saphenous nerve	≤ 3 months preoperatively	No cryoneurolysis	Opioid prescriptions for 90 days	No difference in 90-day opioid use; similar refill rates	Retrospective; short follow-up; confounding by indication
Multicenter prospective registry [20]	2021-2024	174 cryoneurolysis vs 182 controls	Genicular nerves (variable)	≤ 30 days preoperatively	No cryoneurolysis	Pain; opioid use; function (KOOS JR)	Modestly lower pain and improved function at 12 months	Observational design; protocol heterogeneity; residual confounding

Because cryoneurolysis selectively affects sensory rather than motor fibers, quadriceps strength and proprioception are preserved. This motor-sparing profile supports early rehabilitation and allows patients to mobilize safely after surgery, unlike femoral or adductor nerve blocks, which may weaken the leg and increase fall risk [9,10]. The ability to maintain full motor function while achieving extended analgesia is particularly valuable in an opioid-sparing context, as early mobility and improved comfort may further reduce postoperative opioid needs and improve rehabilitation.

Duration of Analgesia and Longer-Term Outcomes

Most studies of cryoneurolysis in TKA have focused on the acute recovery period, typically the first 6 to 12 weeks after surgery, when pain and opioid consumption are highest. Evidence beyond this window is more limited and should be interpreted in the context of the study population and design [8,16]. In a sham-controlled randomized trial of patients with knee osteoarthritis, some of whom were preparing for TKA, cryoneurolysis of the infrapatellar branch of the saphenous nerve was associated with significantly lower pain scores and reduced analgesic use for up to 150 days following treatment [21]. Although this study was not specific to postoperative TKA patients, it provides supportive evidence that cryoneurolysis can produce sustained analgesic effects in knee pain populations, rather than direct evidence of prolonged benefit after TKA.

More directly relevant, a recent multicenter registry study reported that patients who received preoperative cryoneurolysis experienced reduced pain and lower rates of prolonged opioid use during the first postoperative year, as well as modest improvements in functional scores, relative to matched controls [22]. Early postoperative consumption metrics also suggest a time-dependent effect. In a single-center randomized controlled trial, the primary endpoint was cumulative opioid consumption in total daily morphine equivalents from discharge to the six-week follow-up assessment. Although the intention-to-treat analysis did not show a significant difference at six weeks, per-protocol analysis demonstrated significantly lower cumulative opioid consumption with cryoneurolysis at 72 hours, 6 weeks, and 12 weeks [8]. Additionally, registry analyses have shown sustained reduction in opioid utilization at 12 months, lower pain scores over 12 months, and improved Knee Injury and Osteoarthritis Outcome Score for Joint Replacement (KOOS JR), with reduced sleep disturbances [20]. However, functional findings have been less consistent across the broader literature, and pooled comparative data have not demonstrated a statistically significant overall difference in standard functional outcome measures, suggesting that the most reliable signal remains analgesia and opioid reduction, rather than durable functional improvement [19].

Cryoneurolysis and opioid stewardship

A key rationale for integrating cryoneurolysis into TKA pathways is its potential to advance opioid stewardship by curbing the need for postoperative opioids. In an era focused on reducing opioid exposure, especially among surgical patients, cryoneurolysis offers a non-opioid method of pain control that can diminish the demand for analgesic narcotics. There is evidence that TKA patients with poorly controlled acute pain or high initial opioid requirements are at greater risk for chronic opioid use and inferior outcomes [3,4]. By contrast, patients treated with cryoneurolysis have consistently demonstrated lower inpatient opioid consumption in the immediate postoperative period, a reduction that may improve comfort and lessen opioid-related adverse effects [16,17]. Given broader arthroplasty evidence linking greater early opioid exposure with prolonged postoperative use, reduced early opioid requirements with cryoneurolysis may support opioid stewardship; however, direct evidence that cryoneurolysis itself reduces rates of persistent postoperative opioid use after TKA remains limited [20,23,24].

In practice, preliminary data offer a mixed picture on whether cryoneurolysis measurably reduces opioid use beyond the hospital stay. A retrospective review of prescription data for the first 90 days after TKA found no significant difference in total opioid consumption between cryoneurolysis recipients and controls, and approximately 17% of patients in both groups required at least one opioid prescription refill [18]. Among patients who requested a postoperative refill, a greater proportion had received cryoneurolysis; however, this finding should be interpreted cautiously, as it may reflect selection bias, confounding by indication, or lower initial discharge prescribing, rather than failure of cryoneurolysis itself to reduce postoperative opioid requirements [18]. Importantly, this study was limited to a three-month follow-up, so it could not assess differences in longer-term opioid dependence or the total duration of opioid therapy. However, other longitudinal analyses have suggested a possible sustained opioid-sparing effect extending beyond the early recovery period. While these longer-term findings require further validation, they indicate that cryoneurolysis may contribute meaningfully to opioid stewardship when incorporated into a multimodal pain pathway [20,22].

From a clinical implementation standpoint, cryoneurolysis aligns well with modern multimodal analgesia protocols designed to minimize opioid exposure. It can be scheduled in the preoperative period (typically three to seven days before surgery) as an additional component of the patient’s pain management regimen [8]. Ideal candidates may include those with severe knee arthritis pain, patients with high expected postoperative analgesic needs, or individuals with a history of difficulty controlling pain after surgery [10]. By contrast, patients with pre-existing peripheral neuropathy or impaired peripheral circulation may not tolerate cryoneurolysis as well, and are often excluded due to risk of delayed nerve recovery or poor wound healing at the treatment site [10]. When used in appropriate patients, cryoneurolysis is integrated alongside other modalities, such as regional nerve blocks, periarticular local anesthetic options, acetaminophen, nonsteroidal anti-inflammatory drugs (NSAIDs), and neuraxial anesthesia, each addressing pain via different mechanisms [25].

Notably, cryoneurolysis does not obviate the need for standard intraoperative or immediate postoperative analgesia (e.g., many clinicians will still utilize an adductor canal block or periarticular injection during TKA to cover the initial 24-48 hours) [26]. Instead, cryoneurolysis provides an extended pain relief bridge that continues as the effects of shorter-acting modalities wane. In essence, cryoneurolysis can enhance the quality of anesthesia (by providing substantial pain relief) while preserving the quantity of function, thereby supporting a faster recovery with potentially less opioid intake. Surgeons and care teams implementing cryoneurolysis have reported being able to prescribe fewer opioids at discharge for patients who had cryoneurolysis treatment, confident that these patients will have lower pain levels in the first weeks postoperatively [22]. This aligns with current guidelines encouraging minimized opioid prescriptions after joint replacement [27-30]. A comparison of cryoneurolysis with other commonly used perioperative analgesic strategies for TKA is shown in Table 2.

**Table 2:** Comparative perioperative analgesic strategies for total knee arthroplasty The table summarizes the cited literature and practical perioperative considerations qualitatively. Entries reflect the authors’ narrative synthesis of available evidence and clinical implementation factors, rather than direct head-to-head comparative trials or formal quantitative pooling across modalities. Studies included: Mihalko et al. (2021) [8], Hajiaghajani et al. (2026) [19], Hasabo et al. (2022) [31], de Arzuaga et al. (2023) [32], Hussain et al. (2023) [33], Ilfeld et al. (2010) [34], AAOS (2022) [35], Hannon et al. (2022) [36], Capdevila et al. (2005) [37], Dobson et al. (2023) [38], Karam et al. (2021) [39], Li et al. (2021) [40]. RCT: randomized controlled trial; NSAIDs: nonsteroidal anti-inflammatory drugs; US: ultrasound

Modality	Mechanism	Duration	Typical Impact on Early Pain	Typical Opioid-Sparing Effect	Typical Motor Weakness Risk	Practical Complexity	Overall Evidence Base
Cryoneurolysis [8,19]	Targeted freezing of sensory nerves causing reversible axonal degeneration	Weeks to months	Moderate reduction; less effect in first 24-48 h	Moderate; sustained through 6-12 weeks	None	Moderate (preop procedure, specialized equipment)	Moderate; limited RCTs, heterogeneous protocols
Adductor Canal Block [31-33]	Local anesthetic blockade of saphenous nerve/nerve to vastus medialis	12-24 h (single-shot); 48-72 h (continuous)	Strong reduction in first 48 h	Moderate to strong	Low	Moderate (US-guided; catheter management if continuous)	Strong; multiple RCTs and meta-analyses
Femoral Nerve Block [31,34]	Local anesthetic blockade of femoral nerve (sensory + motor)	12-24 h (single-shot); 48-72 h (continuous)	Very strong early pain reduction	Strong (early)	High (quadriceps weakness, fall risk)	Moderate to high (mobilization limits, fall precautions)	Strong; extensive RCT data
Periarticular Injection [35,36]	Intraoperative local anesthetic infiltration ± adjuvants	12-24 h (standard); limited benefit beyond with liposomal formulations	Strong early pain reduction	Moderate to strong	None	Low (intraoperative, surgeon-administered)	Strong; RCTs, meta-analyses, guideline-supported
Continuous Peripheral Nerve Catheter [37,38]	Continuous local anesthetic infusion via indwelling catheter	48-72 h	Very strong during infusion	Strong	Variable (high with femoral; low with adductor canal)	High (catheter care, pumps, monitoring)	Strong; RCT evidence
Systemic Multimodal Analgesia [39,40]	Combined NSAIDs, acetaminophen, corticosteroids ± gabapentinoids	Days to weeks	Moderate alone; strong in combination	Moderate to strong	None	Low to moderate (medication coordination)	Very strong; standard of care

Limitations

Despite the positive trends reported, the current literature on preoperative cryoneurolysis in TKA has important limitations that temper any broad conclusions. First, the number of published studies is relatively small, and many are single-center investigations with modest sample sizes. This limits the generalizability of findings and raises the possibility of selection bias or uncontrolled confounding factors influencing outcomes. There is also considerable heterogeneity in how cryoneurolysis has been applied across studies. Different investigations have targeted different nerve distributions [16,17,19]. Device-specific heterogeneity further complicates interpretation. Most published TKA studies have evaluated the Iovera° system (Pacira BioSciences, Parsippany, New Jersey, USA), whereas at least one randomized trial used a different system, and comparative evidence across cryoneurolysis platforms in the perioperative TKA setting is lacking [9,15,19]. Additionally, various cryoneurolysis devices and protocols have been used, making it difficult to determine whether the benefits observed are generalizable to the technique as a whole or tied to specific equipment and parameters. This procedural heterogeneity complicates direct comparisons between studies and introduces variability in outcomes.

Differences in overall preoperative pain management regimens present another confounder. Some studies enrolled patients in enhanced recovery programs or provided prehabilitation and extensive patient education, whereas others did not standardize these factors [21,22]. Variations in concomitant analgesic use can influence outcomes and make it challenging to isolate the independent contribution of cryoneurolysis. Timing of the cryoneurolysis intervention relative to surgery is also inconsistent in the literature. The optimal timing to maximize efficacy is not yet defined, and this variability adds another layer of uncertainty. Complication reporting is also inconsistent. Although major adverse events have been uncommon in the available TKA literature, detailed characterization of sensory side effects has varied, with some studies explicitly describing dysesthesia, whereas others report only that no complications were observed. Long-term safety surveillance remains limited, so delayed neuropathic sequelae, such as persistent sensory change or neuroma formation, are not well characterized in the TKA-specific literature [17,28,29]. Implementation and learning-curve effects are similarly underreported. One early study suggested only a limited learning curve, whereas later work described protocol optimization related to probe selection, ultrasound-guided nerve identification, and treatment workflow before standardized adoption, suggesting that operator-dependent factors may contribute to heterogeneous outcomes [16,17,30]. Follow-up durations in most reports have been short. As a result, long-term effects on chronic knee pain, sustained opioid use, or functional outcomes beyond the initial recovery period remain largely unknown. Only a minority of studies have included objective measures, such as prescription refill data or serial functional scores, which are crucial for understanding the real-world impact on opioid stewardship and rehabilitation.

Future directions

To address these gaps, future research on cryoneurolysis in TKA should prioritize well-designed, large-scale studies and standardized methodologies. Multicenter randomized controlled trials are particularly needed to validate the efficacy signals observed in early studies under higher levels of evidence. Such trials should use consistent protocols, including standardized nerve mapping, uniformly defined target nerves, prespecified device settings, and a fixed interval between treatment and surgery. Future work should also aim to develop practical patient selection algorithms that identify which patients are most likely to benefit from preoperative cryoneurolysis.

Moreover, future studies must incorporate robust outcome measures that encompass both clinical and patient-centered endpoints. Beyond simply measuring in-hospital pain scores and opioid consumption, studies should track total postoperative opioid usage, time to cessation of opioid therapy, and rates of persistent opioid use at defined intervals. Objective data from pharmacy records or prescription monitoring programs could be particularly enlightening in determining whether cryoneurolysis affects opioid-seeking behavior or refill frequency, in the months after TKA. Concurrently, pain outcomes should be followed over a longer term using validated scales, and functional recovery should be measured with tools such as range-of-motion assessments and patient-reported outcome measures related to knee function and quality of life. Future studies should also incorporate cost-effectiveness and implementation analyses, including effects on length of stay, discharge disposition, readmissions, downstream healthcare utilization, and the overall economic value of adding cryoneurolysis to contemporary multimodal TKA pathways.

Future trials or observational studies might directly compare cryoneurolysis against other regional analgesic techniques, such as single-injection nerve blocks, continuous peripheral nerve block catheters, or extended-release local anesthetic injections, to determine the unique benefits or drawbacks of each. Additionally, research should explore cryoneurolysis in specific subpopulations, such as patients on long-term opioids or those with opioid tolerance. Comparative studies should also evaluate different nerve-targeting strategies and mapping approaches to determine whether certain anatomic targets or patient profiles are associated with a more reliable benefit. By stratifying outcomes by patient characteristics, we can refine selection criteria and personalize the application of cryoneurolysis.

## Conclusions

Preoperative cryoneurolysis is a promising adjunct within multimodal TKA pain pathways, providing prolonged, site-specific, motor-sparing analgesia by inducing reversible axonotmesis in targeted anterior knee sensory nerves, and thereby reducing nociceptive input during the early postoperative period. Current evidence suggests potential benefit for early pain control and reduced inpatient opioid consumption, with some studies also reporting shorter length of stay. Emerging longitudinal and registry data suggest possible benefit beyond the immediate postoperative period, although these findings remain mixed and should be interpreted cautiously.

Clinically, cryoneurolysis should be viewed as complementary rather than substitutive to standard multimodal strategies (e.g., periarticular injection, adductor canal block, acetaminophen, NSAIDs), with the potential to support opioid-sparing recovery while preserving strength and early mobilization. However, heterogeneity in nerve targets, timing, devices, and co-interventions, along with limited sample sizes and short follow-up in many studies, constrains broad generalizability and leaves the magnitude of durable functional benefit uncertain. Larger, standardized, multicenter trials with objective opioid endpoints (total morphine equivalents, refill rates, time to cessation, persistent use), and validated longer-term pain and function measures are needed to define optimal protocols, refine patient selection, and clarify cryoneurolysis’s role in routine TKA care and opioid stewardship.
